# Evaluation of long-term safety profile of an EU-GMP certified *Cannabis sativa* L. strain in a naturally aging preclinical model

**DOI:** 10.3389/fphar.2025.1716366

**Published:** 2025-11-20

**Authors:** Gabriela-Dumitrita Stanciu, Ivona Costachescu, Daniela-Carmen Ababei, Andrei Szilagyi, Raluca-Maria Gogu, Vlad-Constantin Craciun, Andrei-Daniel Timofte, Irina-Draga Caruntu, Cristina-Elena Dobre, Bogdan-Ionel Tamba

**Affiliations:** 1 Advanced Research and Development Center for Experimental Medicine ”Prof. Ostin C. Mungiu” - CEMEX, Grigore T. Popa University of Medicine and Pharmacy Iasi, Iasi, Romania; 2 Pharmacodynamics and Clinical Pharmacy Department, Grigore T. Popa University of Medicine and Pharmacy Iasi, Iasi, Romania; 3 Department of Computer Science, “Alexandru Ioan Cuza” University of Iasi, Iasi, Romania; 4 Department of Morphofunctional Sciences 1, Grigore T. Popa University of Medicine and Pharmacy Iasi, Iasi, Romania; 5 Socola Institute of Psychiatry Iasi, Iasi, Romania; 6 Department of Pharmacology, Clinical Pharmacology and Algesiology, Grigore T. Popa University of Medicine and Pharmacy Iasi, Iasi, Romania

**Keywords:** inflammaging, natural aging, preclinical model, *Cannabis sativa L.*, EU-GMP certification, chronic intermittent therapy, long-term safety profile

## Abstract

Aging is characterized in part by chronic, low-grade inflammation, a major driver of cognitive decline, metabolic imbalance and organ dysfunction. Despite its central role in age-related morbidity, pharmacological strategies with well-defined long-term safety profiles remain limited. Phytocannabinoids have been proposed as modulators of neuroinflammatory and metabolic pathways, but their chronic safety during natural aging is poorly characterized. Our team has previously reported the acute and 28-day repeated-dose toxicity profile of an EU-GMP certified *Cannabis sativa* L. strain (Cannabixir® Medium Flos). Here, we extend this work by assessing its long-term safety in a naturally aging preclinical model. Mature to older mice received chronic, intermittent administration of Cannabixir® Medium Flos (2.5, 5, and 10 mg/kg), defined as daily weekday dosing for 3 or 6 months. Clinical and histopathological evaluations were conducted with a focus on systemic and central nervous system safety. Chronic administration was well tolerated across all doses and durations. Body weight remained stable despite increased food intake. Respiratory quotient values were preserved and close to 1 across all groups. Histological analyses confirmed preserved neuronal and glial architecture with no evidence of central nervous system injury or other organ-level toxicity. Long-term, intermittent Cannabixir® Medium Flos administration was well tolerated in naturally aged mice, with no adverse effects on systemic physiology or central nervous system integrity. Together with prior acute and sub-chronic toxicity data, these findings provide robust evidence supporting the long-term safety of EU-GMP certified *Cannabis sativa* L. strain in the context of aging.

## 1 Introduction

Aging is a complex, multifactorial process characterized by progressive loss of physiological integrity, which leads to impaired function and increased vulnerability to chronic disease (Li et al., 2023). A central feature of aging is the presence of chronic, low-grade inflammation, commonly referred to as “inflammaging” - which arises from cumulative cellular and molecular damage, persistent activation of innate immunity and impaired resolution of inflammatory responses (Franck et al., 2025). This persistent inflammatory state contributes to cognitive decline, metabolic imbalance, progressive organ dysfunction and is compounded by the accumulation of senescent cells, dysregulated immune responses and increased oxidative stress (Partridge et al., 2018; López-Otín et al., 2023). Collectively, these age-associated changes compromise physiological resilience, reduce regenerative capacity and accelerate the transition toward frailty, multimorbidity and decreased healthspan (DiLoreto and Murphy, 2015; Tartiere et al., 2024).

Although major progress has been made in describing the biological hallmarks of aging (López-Otín et al., 2023), therapeutic strategies that can safely attenuate these processes remain limited. Conventional pharmacological interventions generally target single molecular pathway, such as mechanistic target of rapamycin (mTOR), sirtuins or inflammatory cytokines are often accompanied by adverse effects or limited translational efficacy (Hassani et al., 2022; Espinoza et al., 2023; Petr et al., 2024). By contrast, naturally derived bioactive compounds offer a broader spectrum of action, often engaging multiple pathways simultaneously, while maintaining favorable safety profiles (Bilkei-Gorzo et al., 2024). Polyphenols, flavonoids and terpenoids are prominent examples of pleiotropic agents investigated for their anti-inflammatory, antioxidant and metabolic regulatory properties (Song and Zhang, 2023; Nowak-Perlak et al., 2025).

Within this context, phytocannabinoids, the bioactive constituents of *Cannabis sativa* plant have emerged as promising modulators of several hallmarks of aging. In preclinical models, they have been shown to influence neuroinflammatory signaling, mitochondrial bioenergetics, proteostasis and redox balance (Dash et al., 2021; Kletkiewicz et al., 2024; Blebea et al., 2024; Wang and Arnold, 2024). Their pleiotropy derives largely from engagement of the endocannabinoid system, a neuromodulatory network comprising cannabinoid receptors type 1 (CB1) and 2 (CB2), endogenous ligands such as anandamide and 2-AG, and associated metabolic enzymes (Nain et al., 2025). CB1 receptors, predominantly expressed in the central nervous system, regulate synaptic plasticity, energy metabolism and mitochondrial function, while CB2 receptors, enriched in immune and glial cells, attenuate microglial activation and cytokine release, thereby limiting chronic neuroinflammation (Rezende et al., 2023). Beyond direct receptor-mediated actions, phytocannabinoids also interact with other signaling systems—including peroxisome proliferator-activated receptors (PPARs), transient receptor potential (TRP) channels and serotonergic receptors—expanding their potential to modulate metabolic homeostasis and stress responses (Komorowska-Müller et al., 2023; Bilkei-Gorzo et al., 2024).

Importantly, the endocannabinoid system itself undergoes profound remodeling with aging, including reduced endocannabinoid tone, altered receptor expression and impaired signaling efficiency, changes that correlate with increased vulnerability to inflammation, metabolic imbalance, and neurodegeneration (Bilkei-Gorzo, 2012; Koch et al., 2020). These age-related alterations highlight the importance of evaluating the long-term safety of cannabinoid-based interventions in naturally aging bodies.

To date, most preclinical studies have focused on acute or short-term cannabinoid exposure, often in young (Von Gunten et al., 2025) or experimentally injured models (Stanciu et al., 2024), or using heterogeneous and non-standardized preparations (Zuo et al., 2022). Such designs limit reproducibility and translational relevance, providing minimal insight into the chronic safety of phytocannabinoid interventions during natural aging.

Methodological rigor and standardized preparations are therefore critical. European Union–Good Manufacturing Practice (EU-GMP) certification ensures consistent phytochemical composition and purity, prerequisites for reproducible pharmacological and toxicological investigations. Chronic, intermittent administration models, mimicking weekday dosing in humans, offer a physiologically relevant framework to assess cumulative safety in long-term aging studies.

Based on these observations and to extend our previous studies on the acute and repeated-dose toxicity profile of an EU-GMP certified *Cannabis sativa L.* strain (Cannabixir® Medium Flos) (Filipiuc et al., 2023; Stanciu et al., 2024), the present study evaluated the long-term safety of chronic (3 or 6 months), intermittent administration at doses of 2.5, 5, and 10 mg/kg in naturally aging mice. Comprehensive clinical and histopathological assessments were conducted to determine systemic and central nervous system safety. By extending the exposure duration beyond acute and sub-chronic studies, this approach provides a robust platform to establish the long-term safety profile of standardized phytocannabinoid approaches in the context of natural aging.

## 2 Materials and methods

### 2.1 Animal care

Adult male CD1 mice from Cantacuzino Institute, Bucharest, Romania were used in this study. Animals entered the protocol at 40 weeks of age, with endpoints evaluated at 13 and 16 months, corresponding to early- and mid-aging stages. Mice were maintained in individually ventilated cages (IVCs) within the Advanced Research and Development Center for Experimental Medicine “Prof. Ostin C. Mungiu” - CEMEX, under controlled environmental conditions (temperature 20 °C ± 4 °C, relative humidity 50% ± 5% and a 12 h light/dark cycle). Standard laboratory chow and water were provided *ad libitum*.

All experimental procedures were conducted in accordance with Directive 2010/63/EU of the European Parliament and national legislation (Law no. 43/2014) on the protection of animals used for scientific purposes. The study protocol was reviewed and approved by the Ethical Committee of “Grigore T. Popa” University of Medicine and Pharmacy Iaşi (approval no. 187/17.05.2022), and subsequently authorized by the National Sanitary Veterinary and Food Safety Authority (approval no. 57/17.06.2022).

### 2.2 Reagents and study design

The *Cannabis sativa* L. phytocomplex used in this study (Cannabixir® Medium Flos; PZN: 7,001,905; Cansativa GmbH, Mörfelden-Walldorf, Germany) was produced under EU-GMP certification, ensuring standardized quality and purity. According to the manufacturer’s Certificate of Analysis (No. POO5840/11.05.2022), the preparation contained 15.6% Δ^9^-tetrahydrocannabinol (THC) and <1% cannabidiol (CBD). Manufacturing was carried out in compliance with EU Good Manufacturing Practice, the European Pharmacopoeia and the Notice on the German Pharmacopoeia 2017 issued by the Federal Institute for Drugs and Medical Devices (BfArM) on 5 May 2017. Analytical testing was performed in a facility licensed under Section 13 of the German Medicinal Products Act.

Dried *Cannabis* inflorescences were finely ground using an RM 200 electrical mortar grinder (Retsch GmbH, Haan, Germany) and sieved through a 125 μm mesh (BSS Mesh No. 120) to obtain a homogeneous powder, which was suspended in 0.1% sodium carboxymethyl cellulose (CMC-Na). Treatments were administered once daily by oral gavage, consisting of Cannabixir® Medium Flos at doses of 2.5, 5 or 10 mg/kg body weight or vehicle (0.1% CMC-Na) delivered at 0.5 mL/100 g body weight in accordance with murine safety guidelines. Dose selection was guided by LD_50_ values and the favorable pharmacokinetic profile previously reported (Filipiuc et al., 2023). While the inclusion of higher concentrations could have expanded the pharmacodynamic characterization, the selected doses were chosen to balance safety and therapeutic assessment in this preclinical aging model.

A total of 80 mice were randomly allocated to two main treatment-duration groups: G1 (3 months) and G2 (6 months). Each group was further divided into four subgroups (n = 10 per subgroup). Three-month treatment group (G1): control (GM3) and Cannabixir® at 2.5 mg/kg (GC3a), 5 mg/kg (GC3b) or 10 mg/kg (GC3c). Six-month treatment group (G2): control (GM6) and Cannabixir® at 2.5 mg/kg (GC6a), 5 mg/kg (GC6b) or 10 mg/kg (GC6c). Randomization was performed using a computer-generated sequence implemented in Python, assigning each mouse a unique identifier to ensure equal probability of allocation and minimize bias.

To model chronic intermittent cannabinoid exposure (Lamarque et al., 2001; Mouro et al., 2018), mice received treatment for 5 consecutive days followed by a 2-day drug-free interval, repeated over a total of 90 or 180 days. Orally administered THC and CBD are largely metabolized within 24–48 h and have a short plasma half-life (Filipiuc et al., 2023). The drug-free interval was included to simulate intermittent exposure patterns observed in humans, maintain biological relevance, and avoid continuous receptor overstimulation, rather than to prevent accumulation of the compounds. This regimen has been shown in prior preclinical studies to minimize toxicity while preserving the pharmacodynamic effects of chronic intermittent administration (Copeland et al., 2013; Lovelace et al., 2015).

### 2.3 Metabolic profiling

The mice were individually placed in the calorimetry chambers and allowed to acclimate for 2 h prior to measurements. Following acclimation, respiratory metabolism (oxygen consumption, VO_2_; carbon dioxide production, VCO_2_), food and water intake, as well as locomotor and rearing activity were continuously recorded for 24 h using the Oxylet Pro System- Physiocage (PanLab/Harvard Apparatus, Barcelona, Spain). Data acquisition and analysis were performed with the METABOLISM software (version 3.0, Harvard Apparatus, Spain). For the 3-month treatment groups, measurements were performed at the beginning and at the end of the treatment period, whereas for the 6-month groups, measurements were taken at the beginning, midpoint and end.

### 2.4 Biochemical and histological analyses of collected samples

At the end of the treatment period, animals were anesthetized with an overdose of isoflurane (4%). While under deep anesthesia, approximately 1 mL of terminal blood was collected by cardiac puncture into clot activator vacutainer tubes for biochemical analysis. Death was subsequently ensured by cervical dislocation, in compliance with the ARRIVE guidelines, European Directive 2010/63/EU and AVMA Guidelines for the Euthanasia of Animals (Percie du Sert et al., 2020; European Parliament Council of the EU, 2010; Leary and American Veterinary Medical Association, 2020). A full necropsy was then conducted, including thorough inspection of the external body surface, all orifices and the internal cavities (cranial, thoracic and abdominal), along with their contents. The brain was excised and immersed in 10% neutral-buffered formalin (prepared from 36% stock solution; VWR Chemicals, Radnor, PA, USA) for 24–48 h. Coronal sections were obtained at two levels: through the caudal diencephalon and the cerebellum. The sections were processed according to standard tissue processing protocol by paraffin embedding. The paraffin blocks were sectioned at 4 μm, and the obtained sections were stained with H&E stain. All slides were scanned with the Aperio Leica AT2 scanner (Leica Biosystems, Nussloch, Germany). Histological assessment was performed on digitized images by an experienced pathologist blinded to treatment allocation, ensuring objective qualitative and quantitative evaluation.

The morphological analysis was focused on the following areas of interest: the dentate gyrus and CA3 subfield at the hippocampus; the substantia nigra pars reticulata–part of the basal ganglia; the cerebellar molecular layer. These regions were selected based on the presence of cannabinoid receptors, as reported in the literature (Herkenbaum et al., 1991).

Qualitatively, the microscopic examination concerned the histological aspects of neurons, glial cells and myelinated areas, while also seeking to identify other associated lesions. For the neuronal component, we analyzed the following morphological changes specific to neuronal lesions: degeneration, necrosis, vacuolation, chromatolysis and hypertrophy. For the glial component, the assessed changes were: astrocytosis/gliosis and oligodendrocyte degeneration. The monitored myelin lesions were vacuolation or spongiosis. During the microscopic examination, we also paid attention to associated lesions that could potentially be present in the brain: edema, hemorrhage or mineralization. Finally, we assessed the presence of immune-inflammatory infiltrates.

Quantitatively, using the computer-assisted image analysis facilities of the Aperio Scan Scope Console software (Leica Biosystems, Nussloch, Germany), we quantified, for each experimental animal in each subgroup, the number of rows of neurons in the molecular layer of the dentate gyrus and the pyramidal layer of CA3 (Boss et al., 1985; Ozsan et al., 2025), as well as the neuronal and glial density in the substantia nigra pars reticulata area by applying a counting frame to a 250 × 250 microns region of interest, yielding a total analysis area of 0.0625 mm^2^.

To evaluate potential effects of the therapy on primary organs involved in drug metabolism, serum samples were analyzed for multiple biochemical parameters. Blood collected in vacutainer tubes was centrifuged at 1,500 *g* for 15 min at 4 °C within 30 min of collection. Serum was then assessed using an ACCENT-200 Analyzer (PZ Cormay, Lomianki, Poland). The analysed parameters included creatinine (CRE), alanine aminotransferase (ALT), aspartate aminotransferase (AST), total cholesterol (CHOL), glucose (GLU), albumin (ALB), alkaline phosphatase (ALP), total protein (TP), urea (UREA), total bile acids (TBIL), triglyceride (TRG), low-density lipoprotein (LDL cholesterol), high-density lipoprotein (HDL cholesterol).

### 2.5 Statistical analysis

Statistical analyses were performed using Python 3.11.9 with the pandas, matplotlib, bioinfokit, scipy, and statsmodels libraries. Data normality was assessed using the Shapiro–Wilk test. For datasets with normal distribution and homogeneity of variances, group differences were analyzed using one-way ANOVA, followed by t-tests for pairwise comparisons. For non-normally distributed data, the Kruskal–Wallis rank-sum test was applied, followed by Mann–Whitney U tests for pairwise comparisons. Statistical significance thresholds were defined as: p < 0.05 (*), p < 0.01 (**) and p < 0.001 (***). Results are reported as group mean ± standard error of the mean (SEM), unless otherwise specified. Graphical representations illustrate the results of a one-way or two-way ANOVA, highlighting the interaction between age (3 vs. 6 months) and treatment groups. Groups are defined as follows: GM–control groups, GCa–Cannabixir® 2.5 mg/kg, GCb–Cannabixir® 5 mg/kg, and GCc–Cannabixir® 10 mg/kg.

## 3 Results

### 3.1 Evaluation of general health status and energy metabolism using indirect calorimetry

**FIGURE 1:**
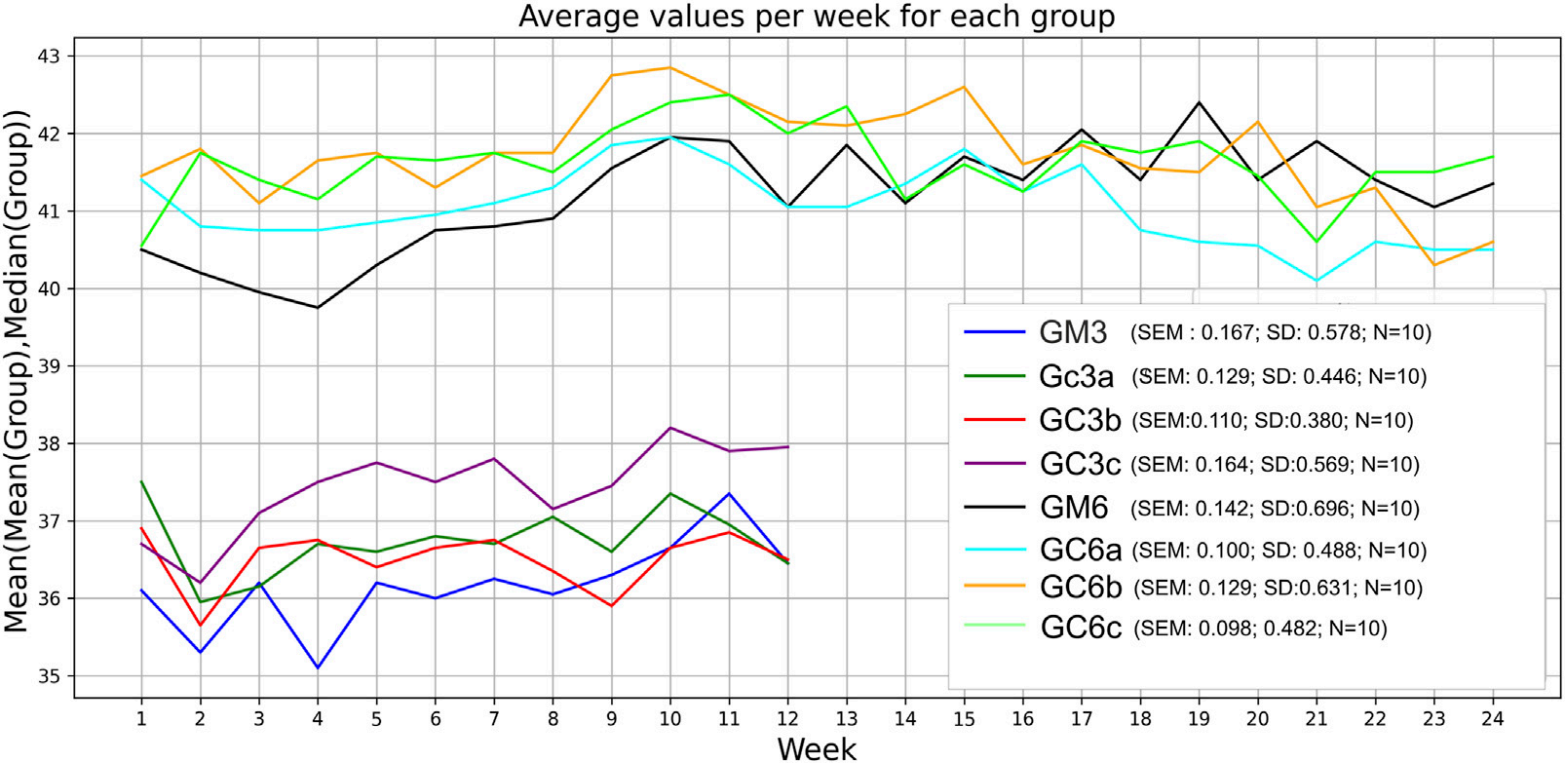
Effect of the therapy on weight gain. Body weight of all animals in each group was monitored weekly during the 3-month or 6-month treatment period. Data are expressed as group mean ± standard error of the mean (SEM). The values correspond to the mean values calculated for each week (x-axis) within the respective experimental group (sub-plot). Significance codes: *p < 0.05; **p < 0.01; ***p < 0.001. The experimental groups were defined as follows: 3-month treatment groups: control (GM3) and Cannabixir® Medium Flos at 2.5 mg/kg (GC3a), 5 mg/kg (GC3b) or 10 mg/kg (GC3c); six-month treatment groups: control (GM6) and Cannabixir® Medium Flos at 2.5 mg/kg (GC6a), 5 mg/kg (GC6b) or 10 mg/kg (GC6c).

Mice received Cannabixir® Medium Flos therapy for 3 or 6 months at doses of 2.5, 5, or 10 mg/kg while maintained on a standard diet. No statistically significant differences in body weight were observed between treated and control animals at any dose or time point. Groups GM and GCc exhibited an ascending trend, with GM6 increasing by 1.5 g and GM3 by 0.8 g, and GC6c increasing by 1.8 g and GC3a by 1.2 g. In contrast, groups GCa and GCb showed a descending trend, with GC6a decreasing by 0.8 g and GC3a by 1.8 g, and GC6b decreasing by 0.7 g and GC3b by 0.3 g. Weight trajectories were not strictly monotonic, with fluctuations occurring during the first 1–1.5 months and occasional local peaks or troughs before reaching the final measurement. These patterns are illustrated in Figure 1, which presents combined mean–median values for all groups. In both the 3- and 6-month groups, the first peak or trough occurred early, within roughly the first 30% of the 3-month interval and before 50% of the 6-month interval.

**FIGURE 2:**
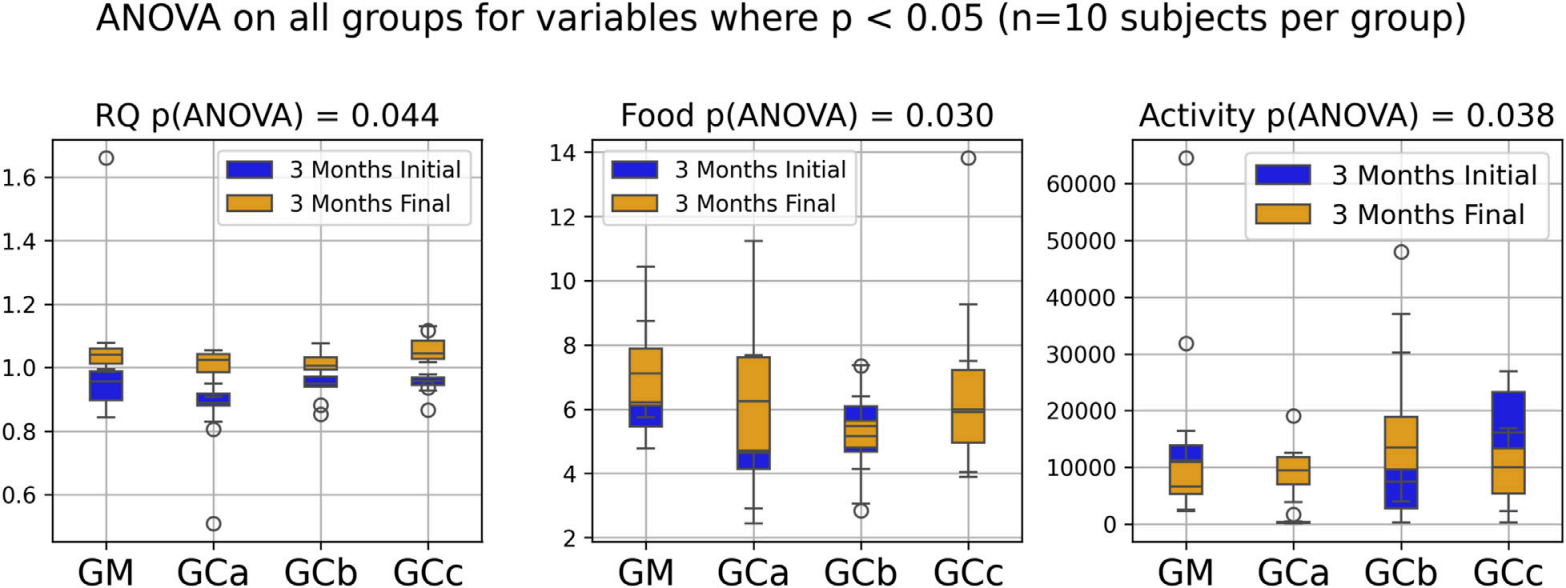
Indirect calorimetry assessment of energy metabolism in healthy, naturally aged mice following 3 months of chronic intermittent Cannabixir® Medium Flos therapy. Oxygen consumption (VO_2_), carbon dioxide production (VCO_2_), respiratory quotient (RQ) or respiratory exchange ratio were continuously recorded over a 24-h period using Oxylet Pro System- Physiocage (PanLab/Harvard Apparatus, Barcelona, Spain). Locomotor activity, food and water intake were recorded simultaneously. The presented plots compare initial and final experimental measurements for RQ, food intake and activity, highlighting only those with statistically significant differences according to ANOVA (p < 0.05). Significance codes: *p < 0.05; **p < 0.01; ***p < 0.001. The experimental groups were defined as follows: 3-month treatment groups: control (GM) and Cannabixir® Medium Flos at 2.5 mg/kg (GCa), 5 mg/kg (GCb) or 10 mg/kg (GCc).

**FIGURE 3:**
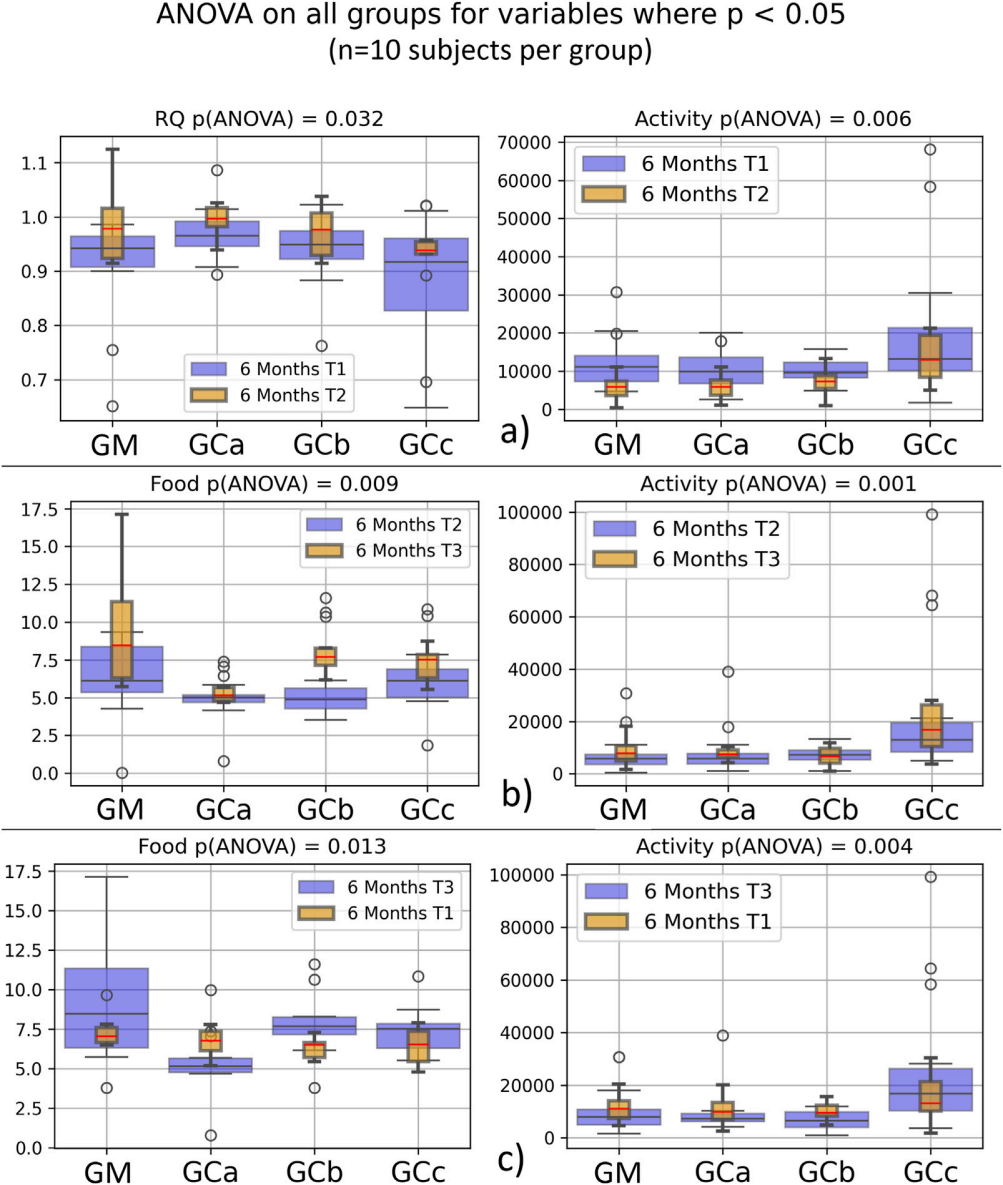
Energy metabolism in healthy, naturally aged mice after 6 months of chronic intermittent Cannabixir® Medium Flos treatment assessed by indirect calorimetry. Oxygen consumption (VO2), carbon dioxide production (VCO2), respiratory quotient (RQ) or respiratory exchange ratio were continuously recorded over a 24‐h period using Oxylet Pro System‐ Physiocage (PanLab/Harvard Apparatus, Barcelona, Spain). Locomotor activity, food and water intake were recorded simultaneously. Sub‐plots show paired variables from T1–T3 datasets, with significant differences (p < 0.05) for Activity (a–c), Food (b,c) and RQ (c). Data were collected at three time points -baseline (T1), midpoint (T2), and endpoint (T3) to monitor temporal changes during treatment. Significance codes: *p < 0.05; **p < 0.01; ***p < 0.001. The experimental groups were defined as follows: six-month treatment groups: control (GM) and Cannabixir® Medium Flos at 2.5 mg/kg (GCa), 5 mg/kg (GCb) or 10 mg/kg (GCc); T1–T3 datasets.

Respiratory metabolism, assessed through oxygen consumption (VO_2_) and carbon dioxide production (VCO_2_), did not exhibit statistically significant alterations in any of the experimental groups throughout the study period. Interestingly, the respiratory quotient (RQ), or respiratory exchange ratio, remained consistently close to 1 across all groups (Figures 2, 3), indicating a predominance of carbohydrate utilization, irrespective of the duration of therapy or the administered compound dose (p = 0.044 for G1 group; p = 0.031 for G2 group).

Food intake showed a statistically significant increase during the experimental period (p < 0.05), suggesting an adaptive metabolic response to therapy administration (Figures 2, 3). In contrast, spontaneous locomotor activity demonstrated a progressive decrease, with the reduction becoming more pronounced as the duration of therapy increased (Figures 2, 3).

### 3.2 Effects of Cannabixir® medium flos on mouse brain neuronal and glial histology and serum biochemical parameters

Qualitative microscopic examination revealed similar findings across all subgroups within the G1 and G2 groups. The histoarchitecture of the dentate gyrus, CA3 subfield of the hippocampus, substantia nigra pars reticulata and cerebellum was preserved.

**FIGURE 4:**
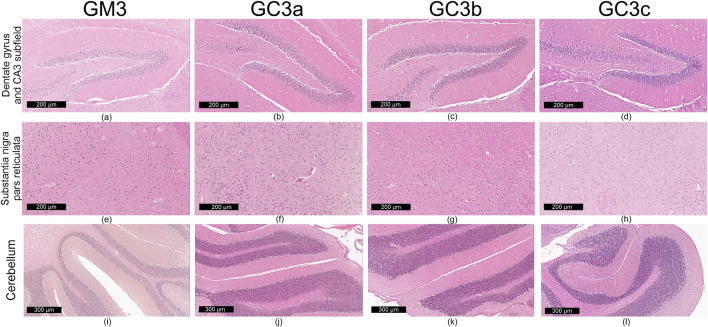
Representative images (n = 10 per group) showing normal histoarchitecture in dentate gyrus and CA3 subfield **(a–d)** neuronal and glial cells in substantia nigra–pars reticulata **(e–h)** and microscopic integrity of cerebellar organization **(i–l)** in the mice brain–in 3-month treatment groups (G1): control (GM3) and Cannabixir® at 2.5 mg/kg (GC3a), 5 mg/kg (GC3b) or 10 mg/kg (GC3c).

**FIGURE 5:**
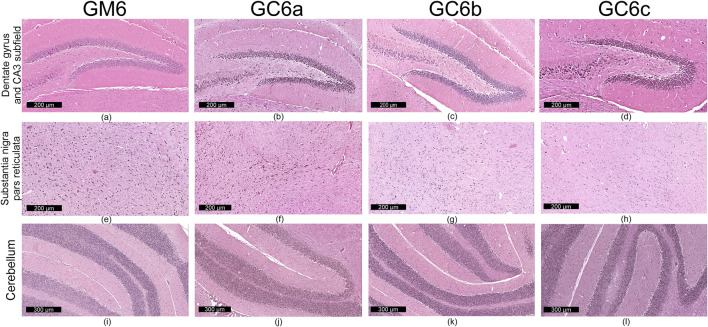
Representative images (n = 10 per group) showing normal histoarchitecture in dentate gyrus and CA3 subfield **(a–d)** neuronal and glial cells in substantia nigra–pars reticulata **(e–h)** and microscopic integrity of cerebellar organization **(i–l)** in the mice brain - six-month treatment groups (G2): control (GM6) and Cannabixir® at 2.5 mg/kg (GC6a), 5 mg/kg (GC6b) or 10 mg/kg (GC6c).

The dental gyrus had a normal structure with a 3-layer organization: the inner polymorphic layer (consisting in mossy cells and interneurons–basket cells and chandelier cells and dendrites), the middle granular layer (consisting in several rows of densely, tightly packed granule neurons) and the outer molecular layers (relatively cell-free and rich in the dendrites of granule cells and perforant path fibers) (Figures 4a–d; Figures 5a–d).

The CA3 subfield presented the typical arrangement consisting in 5 layers: the outer alveus layer (containing axons of CA3 pyramidal cells that enter the fimbria and fornix); the stratum oriens (consisting mainly of axons, dendrites and interneurons); the middle stratum pyramidale (formed by densely, clustered pyramidal cell bodies); the stratum radiatum (formed by axons and dendrites); the inner stratum lacunosum-moleculare (consisting in axons, dendrites, glial cells and a sparse neuronal population) (Figures 4a–d; Figures 5a–d).

In the substantia nigra pars reticulata, we identified fewer neurons grouped in clusters compared to the pars compacta and glial cells; the neurons, which were large and had extensions with very few branches, had reduced amounts of melanin pigment in the cytoplasm (Figures 4e–h; Figures 5e–h). The nerve fibers, which were extremely abundant, had the specific arrangement of a dense network.

The cerebellum showed a distinct organization through the succession of 3 cell layers, from the outside to the inside: the external molecular layer, the Purkinje cell layer and the internal granular layer (Figures 4i–l; Figures 5i–l).

Qualitative analysis of nervous tissue in the dentate gyrus, CA3 subfield and substantia nigra pars reticulata revealed no changes indicative of neuronal and/or glial histological alterations, myelin lesions or other associated lesions (Figures 4a–l; Figures 5a–l).

In all examined areas, the neuronal component did not show: increased size suggesting hypertrophy; contraction, hypereosinophilia and nuclear pyknosis indicating a degenerative process; homogeneous eosinophilic cytoplasm with nuclear fragmentation–morphological features specific to necrosis (Figures 4a–d; Figures 5a–d). We did not find vacuolization with intracellular vacuoles or chromatolysis expressed by swollen perikaryon and eccentric nucleus. In the cerebellum, we noted the presence of edema adjacent to Purkinje neurons in subgroups GC6b and GC6c comprised in G2 group (Figures 5j,k)– this morphological change being absent in all subgroups within G1 group (Figures 4j,k) and in subgroups GM6 and GC6a from G2 group (Figures 5i,j). In substantia nigra pars reticulata, the glial component was characterized by astrocytosis or gliosis mirrored by hyperplasia of glial cells, but oligodendrocyte degeneration consisting in loss or damage of these glial cells was absent (Figures 4e–h; Figures 5e–h).

In all examined areas, the white matter did not display lesions of vacuolation or spongiosis, expressed by intramyelinic edema and/or the presence or empty spaces resembling to vacuoles. No edema, hemorrhage due to small vessel rupture with blood extravasation or mineralization mirrored by focal deposition of calcium salts in injured neurons or glia were present. Focally, we found the immune infiltrate, organized in small clusters of lymphocytes, with random distribution.

For each study group and its corresponding subgroups, the quantified data are presented in Table 1. This includes: the number of neuron rows in the molecular layer of the dentate gyrus and in the pyramidal layer of the CA3 subfield, as well as glial density in the substantia nigra pars reticulata (reported as minimum–maximum values). Images in Figures 4a–l and Figures 5a–l sustain the quantitative analysis.

**TABLE 1:** Dynamics of quantitative neuronal and glial variations.

Morphological characteristics	GM3 [N = 10]	GC3a [N = 10]	GC3b [N = 10]	GC3c [N = 10]	GM6 [N = 10]	GC6a [N = 10]	GC6b [N = 10]	GC6c [N = 10]
	Minimum value – maximum value							
Molecular layer – girus dentate*	7-8	7-8	8-9	6-7	7-9	5-9	6-7	6-9
Pyramidal layer – CA3 subfield*	4-5	4-5	4-5	4-5	4-5	4-5	4-5	4-5
Neuronal and glial component - substantia nigra pars reticulata**	50-60	90-120	60-70	50-60	40-50	30-40	40-50	30-50

* - number of rows; ** - number of cells; [N = 10] - each experimental subgroup included 10 animals.

Quantitative analysis of neuronal layer thickness and cell density in dentate gyrus, CA3, and substantia nigra (ROI, 0.0625 mm^2^. The experimental groups were defined as follows: 3-month treatment groups: control (GM3) and Cannabixir® Medium Flos at 2.5 mg/kg (GC3a), 5 mg/kg (GC3b) or 10 mg/kg (GC3c); six-month treatment groups: control (GM6) and Cannabixir® Medium Flos at 2.5 mg/kg (GC6a), 5 mg/kg (GC6b) or 10 mg/kg (GC6c).

**FIGURE 6:**
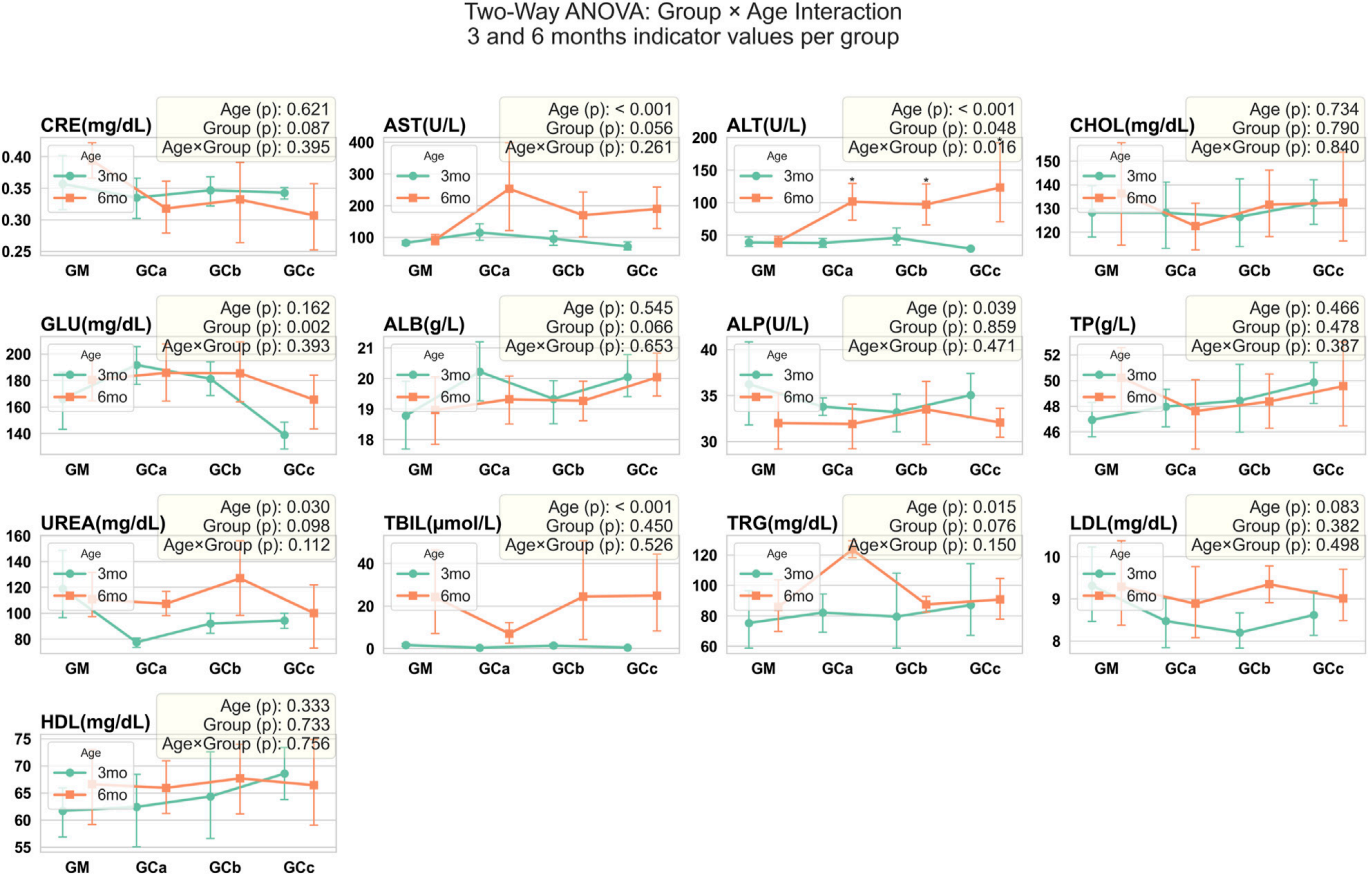
Serum biochemical parameters were assessed in mice following chronic oral administration of Cannabixir® Medium Flos. Data are expressed as mean ± SEM. Statistical analysis was performed using two-way ANOVA to evaluate the effects of Age and Group factors, as well as their interaction (Age × Group). Age refers to treatment exposure duration (3 or 6 months), while Group indicates comparisons within the same exposure duration (intra-group analysis at 3 or 6 months). The Age × Group interaction represents comparisons between treatment groups across different exposure durations (inter-group comparison between 3- and 6-month cohorts). Significant differences are indicated as follows: *p < 0.05; **p < 0.01; ***p < 0.001. CRE, creatinine (mg/dL); ALT, alanine aminotransferase (U/L); AST, aspartate aminotransferase (U/L); CHOL, total cholesterol (mg/dL); GLU, glucose (mg/dL); ALB, albumin (g/L); ALP, alkaline phosphatase (U/L); TP, total protein (g/L); UREA, urea (mg/dL); TBIL, total bile acids (μmol/L); TRG, triglyceride (mg/dL); LDL cholesterol, low-density lipoprotein (mg/dL); HDL cholesterol, high-density lipoprotein (mg/dL); GM, control groups; GCa, Cannabixir® Medium Flos, 2.5 mg/kg groups; GCb, Cannabixir® Medium Flos, 5 mg/kg; GCc, Cannabixir® Medium Flos, 10 mg/kg; therapy period is indicated by line color: 3 months (green) and 6 months (orange).

Biochemical analysis revealed a statistically significant increase in ALT values (p < 0.05) in treated animals compared with controls. For the remaining parameters, trends were observed but did not reach statistical significance. Slight increases in AST, ALP, UREA, ALB, and TRG were seen in some subgroups, while GLU was slightly decreased compared with controls; this decrease was similar in both treatment duration groups (G1 and G2) at the 5 and 10 mg/kg doses. No other consistent differences were detected. Importantly, all values for the parameters, except for the significant ALT elevation, remained within the normal reference ranges for CD-1 mice, indicating that these variations were not clinically relevant (Figure 6).

## 4 Discussions

Cannabis-derived phytocannabinoids, including THC and CBD exert neuromodulatory, anti-inflammatory, and antioxidant effects primarily via the endocannabinoid system (ECS) (Bilkei-Gorzo et al., 2017; Haney, 2022), a central network coordinating cellular signaling, synaptic plasticity and systemic homeostasis (Wang et al., 2003; Wang and Arnold, 2024). CBD has been shown to confer neuroprotection and maintain functional resilience in the aging central nervous system (Ellingson et al., 2021; Winiger et al., 2021), whereas low-dose THC may enhance cognitive performance and attenuate neuroinflammatory processes in aged models (Bilkei-Gorzo et al., 2017; Fihurka et al., 2022; Nain et al., 2025). Age-associated ECS remodeling manifested as reduced endocannabinoid tone and altered receptor expression can increase susceptibility to oxidative stress, synaptic dysfunction and organ impairment (Paradisi et al., 2012; Sallaberry and Astern, 2018). Although phytocannabinoid pharmacology is well-characterized in acute or short-term studies (Kasten et al., 2019; Jenkins et al., 2023), particularly in young or genetically manipulated animals (Bruijnzeel et al., 2019; Stanciu et al., 2024), comprehensive long-term safety data in naturally aging models remain limited, underscoring the need for chronic evaluation.

Consistent with prior preclinical and clinical investigations reporting minimal or no effects of bioactive components of *Cannabis sativa* on body weight (Tudorancea et al., 2025; Stanciu et al., 2024; Filipiuc et al., 2023; Alshaarawy and Anthony, 2019), our findings indicate that administration of Cannabixir® Medium Flos for 3 or 6 months at doses of 2.5, 5 or 10 mg/kg does not significantly alter overall weight gain in mice maintained on a standard diet. Although minor upward or downward fluctuations were observed in specific groups, these changes were modest, transient and lacked a consistent pattern throughout the study period.

Respiratory metabolism, as measured by oxygen consumption (VO_2_) and carbon dioxide production (VCO_2_), remained largely unchanged across all experimental groups, indicating that the therapy did not significantly alter basal energy expenditure. Interestingly, the respiratory quotient (RQ) remained consistently close to 1, suggesting a sustained reliance on carbohydrate metabolism, independent of therapy duration or dose. Food intake showed a modest but statistically significant increase (p < 0.05), whereas spontaneous locomotor activity exhibited a progressive decline with longer treatment. Animals were routinely monitored for general behavioral patterns, including grooming and social interactions, and no abnormal or adverse behaviors were observed. While formal cognitive testing was not conducted, the absence of overt behavioral alterations can be further explained by the pharmacokinetic characteristics of this EU-GMP-certified *Cannabis sativa* L. strain (15.6% THC) previously described by our team (Filipiuc et al., 2023). Psychoactive THC remains undetectable in serum at oral doses up to 600 mg/kg, being replaced by its acidic precursor tetrahydrocannabinolic acid-A (THCA-A), which exhibits markedly reduced blood–brain barrier penetration and a CB1 receptor affinity at least 62-fold lower than THC. At the lower doses used in our study (2.5–10 mg/kg), circulating THC would remain unquantifiable, while THCA-A primarily mediates peripheral effects through transient receptor potential channels and cyclooxygenase 1 and 2 modulation without central CB1 activation. Its short plasma half-life (T_1_/_2_ ≈ 5.3 h) and rapid clearance preclude meaningful accumulation even under chronic administration. Taken together, these pharmacokinetic features strongly support that the observed patterns of stable 5O_2_/VCO_2_, increased caloric intake and reduced locomotor activity reflect adaptive metabolic and behavioral adjustments rather than central or toxic effects, aligning with previous evidence that cannabinoids may influence metabolic parameters in a context-dependent manner (Silvestri and Di Marzo, 2013; Gorelick et al., 2022; Eitan et al., 2024; Costa et al., 2025).

In parallel, biochemical findings provide insight into the systemic effects of Cannabixir® Medium Flos. Although a statistically significant increase in ALT was observed in treated animals, the values remained within the normal physiological range for CD-1 mice, and no corresponding changes were detected in other liver (AST, ALP) or renal markers (UREA, CREA), suggesting that the fluctuation is unlikely to reflect toxicity. Metabolic parameters such as ALB, TRG and GLU also remained within normal ranges. These results align with previous reports indicating a generally favorable safety profile of cannabinoid-based compounds at comparable doses (Filipiuc et al., 2023; Stanciu et al., 2024; Costa et al., 2025). Henderson et al. (2023) noted that hepatic, reproductive and gastrointestinal alterations were relatively rare in CBD studies, while Bookout et al. (2024) reported moderate liver enzyme increases in dogs treated with CBD formulations, reflecting a limited hepatic response. Emerging evidence suggests that THC may also contribute to liver and kidney protection (Pan et al., 2009; Carmona-Hidalgo et al., 2021) and the combination of THC and CBD in our preparation is consistent with the minor, non-adverse ALT changes observed, supporting the interpretation that these changes do not indicate hepatotoxicity. Complementary findings from a short-term (6-week) rat study conducted by our group (Tudorancea et al., 2025) with the same compound formulation showed no significant alterations in systemic cytokine balance, except for a mild, dose-dependent increase in TNF-α at lower dose (6.25 mg/kg), suggesting limited immune modulation rather than inflammation. Collectively, these results reinforce the interpretation that Cannabixir® Medium Flos exerts no overt systemic or organ-specific toxicity under the tested conditions. Future investigations should integrate oxidative stress and inflammatory biomarkers (e.g., malondialdehyde, superoxide dismutase, tumor necrosis factor-alpha and interleukin-6) to further elucidate potential adaptive or protective mechanisms associated with long-term cannabinoid exposure.

Comprehensive histological examination demonstrated that the cytoarchitecture of major brain regions - dentate gyrus, CA3 subfield of the hippocampus, substantia nigra pars reticulata and cerebellum - was largely preserved across all experimental subgroups. The dentate gyrus and CA3 subfield maintained their characteristic three- and five-layer organizations, respectively, indicating that hippocampal networks remained structurally intact (Mortessagne et al., 2024). Likewise, the substantia nigra pars reticulata showed a normal arrangement of neurons and glial elements, and the cerebellum displayed the classical three-layer pattern. Only limited focal changes were observed, including perineuronal edema adjacent to Purkinje cells in the GC6b and GC6c subgroups and mild astrocytic hyperplasia in the substantia nigra pars reticulata, while oligodendrocytes remained unaffected. No morphological markers of neurodegeneration - such as hypertrophy, necrosis, pyknosis, vacuolization, chromatolysis or white matter spongiosis - were detected. Small, randomly distributed lymphocytic clusters were present but without signs of diffuse inflammatory damage. Such discrete alterations may reflect age-related processes, including low-grade neuroinflammation, oxidative stress, impaired neurogenesis or mild neuronal loss (Giannos and Prokopidis, 2022; Lana et al., 2025). Although these age-associated processes are interrelated and can mutually potentiate one another, in our cohort of healthy, naturally aged mice (13–16 months) they did not compromise the global cytoarchitecture or structural integrity of the evaluated neural structures.

Taken together, these results indicate that chronic administration of Cannabixir® Medium Flos at the tested doses does not produce overt structural or cytological injury in central nervous system tissues and supports a favorable histological safety profile. The preserved neuronal and glial organization also raises the possibility of neuroprotective effects in naturally aged mice, potentially mediated through modulation of the endocannabinoid system (Stanciu et al., 2024; Tudorancea et al., 2025). Future mechanistic studies and quantitative analyses will be essential to confirm these observations and to elucidate the pathways underlying such effects.

## 5 Conclusion

Chronic, intermittent administration of Cannabixir® Medium Flos was well tolerated in naturally aged mice, with no observable adverse effects on body mass or central nervous system cytoarchitecture. Histopathological analyses confirmed the preservation of neuronal and glial architecture, supporting a favorable safety profile. Slight elevations in caloric intake, together with a stable respiratory quotient (∼1), indicate that energy homeostasis is maintained via compensatory metabolic mechanisms without perturbations in substrate utilization. These findings suggest the potential for phytocannabinoid-mediated neuroprotection via modulation of the endocannabinoid system, although the precise molecular pathways remain to be elucidated.

Study limitations include the absence of detailed metabolic profiling (hormonal assays) and the exclusive use of healthy aged mice, which may constrain extrapolation to pathological conditions. Despite these limitations, the results provide fundamental data for the long-term safety evaluation of an EU-GMP certified *Cannabis sativa* L. strain in a naturally aging preclinical model. Moreover, the standardized methodology employed including chronic, intermittent administration, use of EU-GMP certified formulations and comprehensive clinical and histopathological assessment establishes a robust preclinical framework for evaluating long-term systemic and central nervous system tolerability of biological therapies in aging or disease models.

## Data Availability

The raw data supporting the conclusions of this article will be made available by the authors, without undue reservation.

## Abbreviations

mTOR, mechanistic target of rapamycin; CB1and CB2, cannabinoid receptors type 1 and type 2; PPARs, peroxisome proliferator-activated receptors; TRP channels, transient receptor potential channels; CMC-Na, carboxymethyl cellulose sodium solution; CRE, creatinine; ALT, alanine aminotransferase; AST, aspartate aminotransferase; CHOL, total cholesterol; GLU, glucose; ALB, albumin; ALP, alkaline phosphatase; TP, total protein; UREA, urea; TBIL, total bile acids; TRG, triglyceride; LDL cholesterol, low-density lipoprotein; HDL cholesterol, high-density lipoprotein.
